# The circadian clock gene *period* extends healthspan in aging *Drosophila melanogaster*

**DOI:** 10.18632/aging.100103

**Published:** 2009-11-19

**Authors:** Natraj Krishnan, Doris Kretzschmar, Kuntol Rakshit, Eileen Chow, Jadwiga M. Giebultowicz

**Affiliations:** ^1^ Department of Zoology, Oregon State University , Corvallis, OR 97331 USA; ^2^ CROET- Oregon Health and Science University, Portland, OR 97239 USA

**Keywords:** oxidative stress, longevity, RING, neurodegeneration, oxidative stress

## Abstract

There is increasing evidence that aging is affected by biological (circadian) clocks - the internal mechanisms that coordinate daily changes in gene expression, physiological functions and behavior with external day/night cycles. Recent data suggest that disruption of the mammalian circadian clock results in accelerated aging and increased age-related pathologies such as cancer; however, the links between loss of daily rhythms and aging are not understood. We sought to determine whether disruption of the circadian clock affects lifespan and healthspan in the model organism Drosophila melanogaster. We examined effects of a null mutation in the circadian clock gene period (per^01^) on the fly healthspan by challenging aging flies with short-term oxidative stress (24h hyperoxia) and investigating their response in terms of  mortality hazard, levels of oxidative damage, and functional senescence. Exposure to 24h hyperoxia during middle age significantly shortened the life expectancy in per^01^ but not in control flies. This homeostatic challenge also led to significantly higher accumulation of oxidative damage in per^01^ flies compared to controls. In addition, aging per^01^ flies showed accelerated functional decline, such as lower climbing ability and increased neuronal degeneration compared to age-matched controls. Together, these data suggest that impaired stress defense pathways may contribute to accelerated aging in the per mutant. In addition, we show that the expression of per gene declines in old wild type flies, suggesting that the circadian regulatory network becomes impaired with age.

## Introduction

Circadian clocks generate daily endogenous rhythms in behavior, physiological functions, and cellular activities, which are coordinated with external day/night cycles [1,2]. Circadian rhythms become impaired with age as evidenced by the dampening of daily oscillations in melatonin and other hormones and the disruption of night-time sleep in aged rodents and humans [3,4,5]. Remarkably, age-associated sleep fragmentation was also reported in *Drosophila melanogaster*[6], suggesting that effects of aging on circadian systems may be evolutionarily conserved.  While aging impairs the circadian systems, there is also evidence that loss of circadian rhythms may, in turn, contribute to aging. Genetic disruption of circadian rhythms by knockout of specific clock genes leads to various age related pathologies and visible signs of premature aging in mice [7,8].  In addition, chronic jet-lag which disrupts the circadian clock, increases mortality in aged mice [9]. As extension of healthspan is of critical importance in aging human population, there is a need to elucidate how strong circadian clocks may support healthy aging.

The mechanisms linking circadian rhythms to the rate of aging and healthspan are not well understood. To address these mechanisms, we investigated whether disruption of the circadian clock affects response to homeostatic challenge and aggravates selected aging biomarkers in the model organism *Drosophila melanogaster*. We used a null mutation in the circadian clock gene *period* (*per^01^*) [10]; this gene is one of the four core clock genes that act in a negative auto-regulatory feedback loop generating daily endogenous rhythms [11,12]. The loss of *per* function disrupts behavioral and molecular rhythms in flies [10,11,13].

To compare lifespan and healthspan in flies with normal or disrupted circadian clock, we measured their ability to maintain ROS homeostasis during aging. We probed the health status of aging flies by exposing them to mild oxidative stress of 24h hyperoxia at increasingchronological ages, followed by assessment of the resulting oxidative damage and mortality hazards. Hyperoxia was chosen as a homeostatic challenge, because it directly leads to ROS production irrespective of age-related changes in food consumption and other physiological parameters [14].

**Figure 1.**
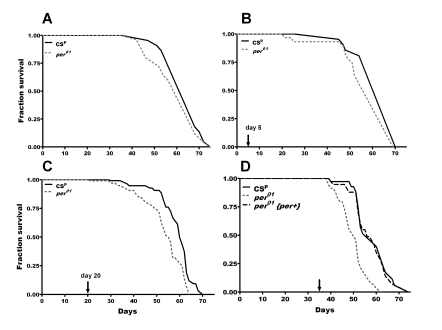
**Lifespan of *per^01^*and CS^p^*D. melanogaster *in normoxia and following 24h hyperoxia at different ages** (marked by arrow in **B-D**). (**A**) In normoxia, there was no significant difference in mean survival curves (p=0.23) (**B**) Hyperoxia on day 5 did not significantly affect longevity or survival curves (p=0.12) (**C**) Hyperoxia on day 20 resulted in a significant reduction (p<0.05) in average survival of *per^01^*flies compared to CS^p^ with significant (p<0.0001) difference in survival curves. (**D**) Hyperoxia on day 35 resulted in more significant reduction (p<0.001) in average lifespan in *per^01^*flies compared to CS^p^ and significant difference in survival curves (p<0.0001). Males with rescued *per *function (*per^01^*{*per^+^*}) treated with hyperoxia on day 35 had average lifespan similar to CS^p ^but significantly different (p<0.001) from *per^01^*mutants.

We report that *per^01^* flies have shortened healthspan as evidenced by their increased mortality hazard in response to homeostatic challenge during aging. This conclusion is also supported by accelerated functional senescence, and increased signs of neurodegeneration in *per* mutants compared to age-matched controls with an intact circadian clock.  In addition, we show that the expression of *per* gene declines with age leading to disruption of the circadian regulatory network in old wild type flies.

## Results

### Short-term oxidative stress shortens the lifespan in per^01^ mutants 

To determine how loss of *per *affects lifespan and healthspan, *per^01^* were backcrossed for 6 generations to Canton S strain, and this control stock was designated as CS^p^.  Under normal laboratory conditions, the longevity of *per^01^* males was similar to CS^p^ controls (Figure 1A, Table 1). However, lifespan was significantly reduced in *per^01^*flies exposed to 24 h hyperoxia in middle age. Hyperoxia on day 20 shortened the average lifespan in *per^01^* mutants by 12% while hyperoxia on day 35 decreased average lifespan of *per^01^* flies by 20% compared to CS^p^ males (Table 1);  survival curves were significantly different in both ages (Figure 1C-D). We also calculated age specific mortality trajectories, and showed that mortality hazard significantly increased after exposure to 24 h hyperoxia on day 20 or 35 in *per^01^* but remained unchanged in CS^p^ males (see Supplementary Figure 1 and Supplementary Table 1). To verify that these effects are indeed linked to the lack of *per* gene function, we tested the lifespan of *per^01^* flies transformed with a wild type copy of *per*, designated as  *per^01^*{*per^+^*}. When flies with rescued *per* function were exposed to hyperoxia on day 35, their  average survival (59 ± 2.0 days) and mortality trajectories were similar to CS^p^ controls, but  significantly different from  *per^01^* mutants (Figure 1D, Supplementary Figure 1D, and Supplementary Table 1). This verified that shortened  lifespan and increased death-risk in *per* mutants are due to the loss of *per* gene. Importantly, exposure to hyperoxia on day 5 did not affect the average lifespan or mortality trajectories of *per^01^* mutants (Figure 1B and Supplementary Figure 1B), demonstrating that hyperoxia sensitivity in these mutants is an age dependent phenotype.

**Table 1.** Average lifespan of CS ^p^ and *per^01^* males exposed to 24h hyperoxia at indicated ages. Values shown with SEM, n denotes the sample size. One-Way ANOVA with Tukey-Kramer multiple comparisons test. Statistical comparison across genotypes * = p<0.05, ** = p<0.001; within genotype, values with different superscripts are significantly different at p<0.05.

**Treatment**	**Genotypes**	
**CS^p^**	***per^01^***
**Normoxia**	61.5 ± 1.8^a^ (*n= 596*)	59.0 ± 1.02^a^ (*n= 640*)
**Hyperoxia day 5**	60.4 ± 0.8^a^ (*n= 447*)	56.9 ± 0.93^b^ (*n= 480*)
**Hyperoxia day 20**	58.4 ± 0.93^a^ (*n= 415*)	51.35 ± 1.07*^c^ (*n= 385*)
**Hyperoxia day 35**	59.5 ± 1.03^a^ (*n = 328*)	47.8 ± 1.68**^c^ (*n= 350*)

### *per^01^* mutants accumulate more oxidative damage in response to stress and during normal aging

**Figure 2.**
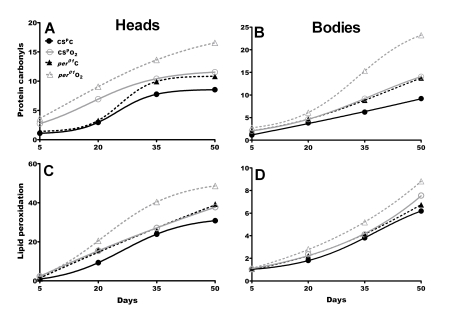
**Oxidative damage accumulates to higher levels in aging *per^01^*flies.** Fold increase was calculated based on day 5 values in CS^p^ males under normoxia (numerical values are shown in Supplementary Table 2 and Supplementary Table 3). **Top**: Protein carbonyls (PC) in heads (**A**) and bodies (**B**) of CS^p^ (solid line) and *per^01^*(broken line) in normoxia (black) and after hyperoxia (gray). PC levels were significantly higher in *per^01^*than in CS^p  ^fly heads on day 35 and 50, and on day 50 in bodies under normoxia. Hyperoxia on day 35 and 50 induced significantly higher PC levels *per^01^*head and bodies compared to CS^p^ age-matched controls. **Bottom**: Lipid peroxidation product 4-HNE in heads (**C**) and bodies (**D**). In normoxia, *per^01^*flies accumulated significantly more 4-HNE in heads and bodies compared to CS^p^ in all ages except day 5. Under hyperoxia, significant increase in 4-HNE accumulation was observed in *per^01^*heads and bodies on day 20, 35 and 50 compared to CS^p^ males. For statistical analysis of PC and HNE data refer to Supplementary Table 2 and Supplementary Table 3.

Given the increased mortality hazard in response to hyperoxia in *per^01^* mutants, we next assessed the levels of oxidative damage incurred after 24 h hyperoxia exposure at the age of 5, 20, 35 and 50 days in both genotypes. Levels of protein carbonyls (PC) and the lipid peroxidation product 4-HNE were measured separately in heads and bodies. Exposure to hyperoxia induced significantly higher (p<0.001) PC levels in* per^01^* than in CS^p ^heads at all ages except day 5 (Figure 2A and Supplementary Table 2). Similar as in heads hyperoxia on day 35 or 50 led to moderate PC increase in CS^p^ bodies and dramatic increase in the bodies of *per^01^* flies (Figure 2B and Supplementary Table 2). Restoring *per*^+^ function in a *per^01^* background resulted in PC content similar as in CS^p^ and significantly lower than in *per^01^*males (Supplementary Table 2). Thus, the loss of *per* function leads to dramatically higher accumulation of PC in *per^01^* flies faced with oxidative challenge. Similar as in the case of mortality hazard this deleterious phenotype is age dependent occurring in middle aged and old flies but not young *per^01^*mutants (Figure 1-2 and Supplementary Figure 1).

The second indicator of oxidative damage, the lipid peroxidation product 4-HNE, was also measured in heads and bodies of CS^p^ and *per^01^* flies. Exposure to hyperoxia on day 35 and 50 significantly increased HNE in *per^01^* heads compared to respective CS^p^ controls (p<0.001) while exposure on day 5 or 20 had no significant effect (Figure 2C and Supplementary Table 3). Similar as in heads, hyperoxia administered on day 35 and 50 induced significantly more HNE in *per^01^* than in CS^p^ bodies, however, the increase was less pronounced than in fly heads (Figure 2C-D). These effects depend on the *per* gene as males with restored *per* function exhibited significantly lower HNE profiles than  *per^01^*males, and similar as those observed in CS^p^ flies  (Supplementary Table 3).

### Aging* per^01^* mutants show greater mobility impairment and neurodegeneration

**Figure 3.**
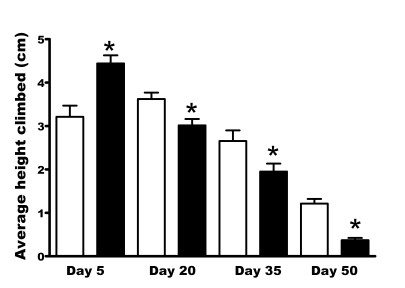
**Vertical mobility deteriorates faster in *per^01^*flies, as demonstrated by the RING assay.** Bars represent mean height climbed (with SEM) in CS^p^ (open bars) and *per^01^*(black bars) males at indicated age. The climbing performance of *per^01^*males on day 5 was significantly higher (p<0.001) compared to CS^p^. With age, a rapid deterioration in climbing performance was noted in *per^01^*flies with mobility being significantly lower (* p<0.001) on day 20, 35, and 50 compared to age-matched CS^p^ controls.

Our data show significantly higher accumulation of oxidative damage even in unchallenged *per^01^* mutants under normoxia compared to age matched controls (Figure 2, Supplementary Table 2, Supplementary Table 3). As oxidative damage is one of the important biomarkers of aging, we asked whether other signs of aging are advanced in *per^01^* mutants. First, we compared age-related locomotor performance between mutant and control flies. We used the RING assay, which utilizes negative geotaxis in *Drosophila* to assess climbing performance [15,16]. We measured climbing ability of *per^01^* and CS^p^ flies aged to day 5, 20, 35 or 50. Surprisingly, 5 day old *per^01^* flies showed significantly higher climbing ability than control flies. In contrast, middle-aged and older *per^01^* males showed significantly impaired climbing ability compared to age-matched controls (Figure 3). The difference was especially dramatic on day 50; at this age the average climbing ability of *per^01^* males was approximately 4 fold lower than in CS^p^ controls. This was partly caused by lack of vertical movement in many *per^01^* flies at this age. The fact that young *per^01^* mutant flies did not show impaired climbing demonstrate that the *period* gene does not affect fly geotaxis *per se*, but rather contributes to impaired  climbing ability in an age-dependent fashion.

**Figure 4.**
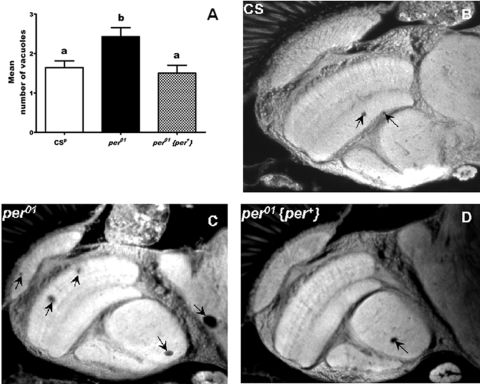
**Neuronal degeneration is accelerated in *per^01^*mutants compared to CS^p^ and flies with restored *per *function (*per^01^*{*per^+^*}) on day 50.** (**A**) Mean number of vacuoles (with SEM) representing neuronal degeneration was significantly higher in *per^01^*mutants compared with wild type CS^p^ and flies with rescued *per*. Bars with different superscripts are significantly different at p<0.05, data based on 10-15 heads for each genotype. (**B-D**) Photomicrographs of representative brain sections of CS^p^, *per^01^*, and *per^01^*{*per^+^*} males. Arrows point to vacuolization.

Another indicator of aging that we tested in *per^01^* flies was the health of their nervous system. As aging  is associated with degenerative morphological changes in the central nervous system,  we examined brain sections from  50 day old  *per^01^*, CS^p^, and *per^01^*{*per^+^*} males. We evaluated number of vacuoles, as they reflect the level of neurodegenerative damage in the brain [17]. Brains of *per^01^* males showed significantly (p<0.05) greater number of vacuoles than control CS^p^ and *per^01^*{*per^+^*} flies with restored *per* function  (Figure 4). These vacuoles, which were found mainly in the neuropils of the optic lobes and the central brain, lead to disrupted neuronal connections. Increased vacuolization in 50 day old *per^01^* flies is consistent with their severely impaired mobility (Figure 3).

### Expression of *per* gene declines significantly with age

**Figure 5.**
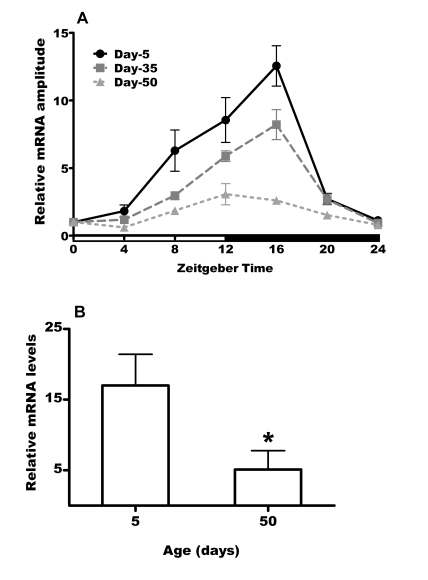
**Expression of *per* mRNA declines with with age in heads of CS^p^ flies**. **(A)** Daily mRNA expression profiles of *per* in day 5, 35 and 50 male heads. White and black horizontal bars mark periods of light and darkness respectively. Values were normalized to *rp49* and calibrated against ZT0 (taken as 1) for each age and represented as mean ± SEM of 3 bioreplicates. **(B)** The peak levels  of *per* mRNA are significantly reduced (* = p<0.05) in 50 day old males compared to young control males. Values are mean ± SEM of 3 bioreplicates.

Since age related functional decline is accelerated in *per^01^* flies compared to flies with normal clock, it was of interest to investigate daily profiles of *per* expression during aging in control CS^p ^flies. Therefore, we used qRT-PCR to measure the expression levels of *per* mRNA extracted from flies collected every 4h for 24h at age 5, 35 and 50 days.  As expected [11], *per *mRNA levels showed daily cycling with lowest levels in the morning and a peak at early night in the heads of young flies (Figure 5A). The levels of *per* between peak and trough changed with a 12-fold amplitude.  This amplitude dampened significantly in 35 day old flies; however, there was still pronounced cycling of *per* mRNA with 8-fold amplitude. A dramatic dampening of *per* oscillation was observed on day 50 with the amplitude reduced to 2-fold. Comparison of the relative *per* mRNA levels at the peak showed significant reduction by ca 70% in 50 day old flies relative to peak expression levels in young flies. Since *per* encodes an essential component of circadian clock,  our data suggest that  the circadian network is severely impaired in old flies.

## Discussion

This study demonstrates healthspan extending role of the clock gene *period* and suggest that functional circadian clocks may prevent premature aging in flies. Research on *Drosophila *has demonstrated that different genetic manipulations and environmental interventions can extend fly lifespan [18].  Less attention has been paid to healthspan, despite that extension of healthspan is of critical importance in aging human population. Here, we show that healthspan can follow different trajectories in flies which have similar lifespan under stress-free laboratory conditions. Healthspan is an important but poorly defined concept, and there is an ongoing debate whether model organisms, such as *Drosophila*, can help to characterize parameters that could detect differences in healthspan [19]. We demonstrate that a relatively mild exogenous stress of 24 h hyperoxia, which revealed health impairment of *per^01 ^*mutant, could be established as a convenient method to probe fly healthspan in a search for mechanisms supporting healthy aging.

Here, we show that healthspan, measured as the ability to respond to homeostatic challenge is reduced in *per^01^* flies. Exposure to mild oxidative stress in middle age significantly shortened life expectancy in *per^01^* flies but, importantly, not in control flies.  The lower capacity of *per^01 ^*mutants to buffer short-term oxidative challenge was linked to greatly increased accumulation of oxidative damage during hyperoxia exposure. Thus, it appears that increased mortality hazard in hyperoxia-exposed *per^01 ^*mutants may be caused by their impaired ability to clear the oxidative damage which is suggested to be one of the major causes of aging [20].

The higher  accrual of oxidative damage observed in *per^01 ^*flies in normoxia and especially after hyperoxia could be influenced by a number of factors, with the primary suspect being higher production of endogenous ROS, which has been reported to increase in clock-disrupted flies [21] and  mice [7]. Whether higher ROS is associated with decreased activity of ROS scavenging en- zymes remains to be determined. While microarray studies suggested that expression of superoxide dismutase and catalase may be controlled by the circadian clock in flies [22], qRT-PCR did not confirm such rhythm for catalase, but demonstrated that catalase activity is significantly lower in young clock-deficient flies [21]. It is currently unknown whether enzymes involved in protein repair are controlled by the circadian clock in animals, although such control was reported in plants [23].  Finally, excessive agglomeration of oxidatively damaged proteins in *per^01 ^*flies could be related to impaired degradation as proteasome activity has been shown to decline with age in flies, and may be inhibited by PC and HNE [24,25]

As in humans, age-related functional declines such as disrupted sleep and decreased mobility are observed in *Drosophila*[6,26]. The negative geotaxis assay revealed significant impairment in climbing ability in aging *per^01^* flies relative to age-matched controls suggesting that lack of *per* impairs physical performance during aging. Importantly, exacerbated mobility decline in *per^01^* flies was associated with increased neuronal degeneration in the brain. Neurodegenerative effects in the form of vacuoles in the neuropil region were observed with higher frequency in 50-day old *per^01^* mutants than in CS^p^ or  *per^01^*{*per^+^*} flies with restored *per* function. The formation of vacuoles was previously linked to oxidative damage and accelerated aging in *Drosophila* with impaired carbonyl reductase gene [27], and in flies with Alzheimer-like phenotypes [28].

Our study suggests that functional circadian rhythms support healthy aging in flies.  PER protein is the essential element of circadian clock and its absence disrupts molecular and cellular rhythms. We reported previously that young wild type flies have daily rhythms in ROS and PC levels, while in  *per^01^* flies levels of these deleterious compounds are significantly higher and arrhythmic [21]. We hypothesize that the circadian clock slows down the accumulation of oxidative damage in aging organisms by synchronizing the activities of enzymes involved in protein homeostasis. For example, microarray studies reported synchronous upregulation of several GST enzymes in flies [29], and it is known that glutathione participates in the conjugation of  oxidized proteins [30].  In the absence of circadian clock, enzymes working in a specific pathway may become dysregulated leading to impaired removal of oxidative damage. However, we cannot exclude the possibility that *per* could affect efficiency of anti-oxidative defense systems independent of its role as a clock component, by acting in a pleiotropic non-circadian manner.

While loss of the circadian rhythms by disruption of the gene* period* accelerates aging, organisms with normal clocks also age. Our data demonstrate that at middle age *per^01^* mutant shows aging phenotypes normally observed in chronologically older wild type flies, suggesting that clock gene activities may decline with age. Indeed, we demonstrate the amplitude of *per* mRNA oscillation is severely dampened in 50 day old flies and levels of *per* mRNA are significantly reduced at late night, when PER acts as essential element of clock negative feedback loop [11].  This suggests that circadian clocks and, consequently circadian rhythms are severely impaired in individuals of advanced age, which is consistent with declining strength of behavioral rhythms reported in aging flies [6]. While factors contributing to the decline of circadian rhythms in flies remain to be elucidated, oxidative stress is likely to be involved. We show here that oxidative damage accumulates to high levels even in wild type aging flies, and a previous report  demonstrated that paraquat-induced  oxidative stress, or decrease in FOXO expression, led to  dampened *per* expression in *Drosophila*[31]. Decline in clock genes with age has been reported in zebrafish [32], rats [33] and most recently in rhesus monkey [34]. The intriguing similarities in the behavior of clock genes during aging between mammals, zebrafish, and flies warrants investigations of the mechanisms causing disruption of the circadian networks. Understanding these mechanisms will help to determine in future whether strong circadian clocks add water to the fountain of youth.

## Experimental procedures

Fly rearing and l ife span analysis. *Drosophila melanogaster* were reared on yeast-cornmeal-molasses-agar diet (35g yeast/l) at 25°C in a 12-hour light/12-hour dark cycles; all experiments were performed 4-8 h after lights-on. The *per^01^* mutant flies  [10] were backcrossed 6 times  to the Canton-S (CS) flies designated as CS^P^. To rescue *per*-function, we used transgenic flies carrying a wild-type copy of *per* (designated as *per*^G^) in a *per^01^* background [35]. Males with two copies of *per*^G^ (*y wper^01^*;{*per^+^*:32.1};+ were crossed with *per^01^*;+;+ females, and F1 males containing one copy of rescue construct designated *per^01^*{*per^+^*} were used. We confirmed their rhythmic locomotor activity indicating rescue of circadian clock function.

To determine lifespan, 3-4 cohorts of 100 flies of each genotype were housed in 16 oz transparent plastic bottles inverted over 60 mm Petri-dishes containing 15 ml of diet. Diet was replaced on alternate days without anesthesia, and mortality was recorded daily.  For hyperoxia exposure, males were transferred from cages to narrow vials with diet in groups of 25, and placed in a Plexiglas chamber filled with oxygen (100% medical grade) flowing at a constant rate (300ml/min) for 24 h. Control flies were transferred to narrow vials as above and kept under normoxia. Hyperoxia-treated and control flies were then either frozen for oxidative damage analysis or returned to cages and monitored for mortality.

Oxidative damage assays. The amount of protein carbonyls was assayed separately in 25 heads and bodies. Carbonyls were quantified after reaction with 2,4-dinitrophenylhydrazine (DNPH) as described previously [21]  at 370 nm in a BioTek Synergy 2 plate reader. Results were expressed as nmol.mg^-1^ protein using an extinction coefficient of 22,000 M^-1^cm^-1^. The lipid peroxidation product 4-hydroxy-2-nonenal (4-HNE) was assayed in heads and bodies by competitive enzyme-linked immunosorbent assay (ELISA) as described [36, 37]. Briefly, free HNE (Alpha Diagnostic, San Antonio, TX, USA) was conjugated to glyceraldehyde-3-phosphate dehydrogenase (GAPDH) protein [38]. Wells in a 96-well plate were coated with 500 ng of HNE-GAPDH protein for 24h at 4°C, washed in PBS-Tween, and blocked with 1% BSA. A standard dose-response curve was developed from serial dilutions of HNE-GAPDH with polyclonal anti-HNE antibody (1:1000; Alpha Diagnostic). For samples, 10 μ;g of protein lysate was mixed with 1:1000 polyclonal rabbit anti-HNE antibody and added to wells in triplicate. Plates were incubated for 1 h, washed with buffer, incubated with 1:5000 secondary anti-rabbit antibody conjugated with horseradish peroxidase, washed, mixed with detection buffer TMB (Alpha Diagnostic), and read at OD 450nm in a BioTek plate reader.

Rapid iterative negative geotaxis (RING) assay. Vertical mobility was assayed using the RING method [15]. Briefly, 3 groups of 25 CS^p^ or *per^01^* flies were transferred into empty narrow vials, which were loaded into the RING apparatus. After 3 minutes rest, the apparatus was rapped sharply on the table three times in rapid succession to initiate a negative geotaxis response. The flies' movements in tubes were videotaped and digital images captured 4 s after initiating the behavior. Five consecutive trials were interspersed with a 30s rest. The climbing performance was calculated and expressed as average height climbed in the 4 s interval. The performance of flies in a single vial was calculated as the average of 5 consecutive trials to generate n = 1.

Neuronal degeneration. Paraffin-embedded sections of heads were used to examine neurodegenerative defects. Fly heads of all genotypes were processed and sectioned in parallel, and microscopic pictures taken at the same level of the brain and the number and volume of vacuoles counted in double-blind experiments using described methods [39, 40].

Quantitative Real-Time PCR. 25 male heads were collected for each time point in triplicate, homogenized in TriReagent (Sigma), and RNA was isolated following manufacturer protocol. Samples were purified using the RNeasy mini kit (Qiagen) with on-column DNAse digestion (Qiagen). Synthesis of cDNA was achieved with Sprint RT Complete kit (Clontech) or iScript cDNA synthesis kit (Biorad). Real-time PCR was performed on Step-One Plus real-time machine (Applied Biosystems) in triplicate under default thermal cycling conditions with a dissociation curve step.  Each reaction contained iTaq SYBR Green Supermix with ROX (Biorad), 0.6-1ng cDNA, 80nM primers (IDT Technologies). Primers sequences are available upon request. Data were analyzed using the standard 2^-∆∆CT^ method normalized to the gene *rp49* and expressed relative to control samples at ZT0.

Statistical analyses. Life span and survival curves were plotted following Kaplan Meier survival analysis and statistical significance of curves assessed using the Log-Rank (Mantel-Cox) and Gehan-Breslow-Wilcoxon test (GraphPad Prism v 5.0).Age-specific mortality was calculated using the Gompertz's model of population aging. Ln values of instantaneous mortality (μ_x_) were plotted against chronological time. Mortality calculations and Gompertz-Makeham maximum likelihood estimates were done using WinModest V1.0.2 [41] and plotted on GraphPad Prism. For statistical analysis of biochemical results three-way ANOVA with post-hoc tests were performed using OpenStat (William G. Miller © 2009). Statistical analysis of locomotor assays was done with one and two-way ANOVA for comparison between ages and genotypes.

## Supplementary information

Supplementary Table 1 Mortality parameters derived from fitted Gompertz-Makeham model and maximum likelihood estimates (MLE). Mortality at age x (μ_x_) is given as μ_x_ = ae^bx^ + *c*, where *a* is the baseline mortality rate (intercept), *b* is the age-dependent increase in mortality (slope), and *c* is the age-independent mortality.

Supplementary Table 2 Protein carbonyl content (nmol.mg ^-1^ protein) in male heads and bodies. Values are Mean ± SEM of 3 separate bioreplicates. Three-way ANOVA with Bonferroni's post-hoc tests was performed for each tissue. Values with different superscripts shown in columns are significantly different at p<0.01. For comparison between genotypes (rows) for each treatment, * = p<0.05 and ** = p<0.001, † = p<0.03 ‡ = p<0.01. Comparison between treatments for each genotype showed significance at p<0.001 in all ages for heads, and on day 35 and 50 for bodies.

Supplementary Table 3 4-HNE content (nmol.mg ^-1^ protein) in male heads and bodies. Values are Mean ± SEM of 3 separate bioreplicates. Three-way ANOVA with Bonferroni's post-hoc tests was performed for each tissue. Values in columns with different superscripts are significantly different at p<0.001. For comparison between genotypes (rows) for each treatment, † = p<0.03, Ψ = p<0.05, ** = p<0.001, *** = p<0.0001. Comparison between treatments for heads showed significant difference (p<0.01) at all ages for *per01*, and on day 35 and 50 for CS^p^. In case of bodies, comparison between treatments showed significance at p<0.01 on day 35 and 50 for both genotypes.

Supplementary Figure 1 Age-specific mortality trajectories (-ln ?x) in normoxia and following 24h hyperoxia at different ages (marked by vertical dotted line) in CS ^p^ and per01 males. Mortality trajectories were plotted using Gompertz-Makeham mortality parameters and smoothed using 2nd order smoothing of 5 neighbors. (**A-B**) Under normoxia and 24h hyperoxia on day 5 no significant difference in mortality trajectories was observed between CS^p^ and *per01* flies. (**C**) 24h hyperoxia on day 20 resulted in significantly different mortality trajectories (p<0.001), with mortality slope of per01 flies becoming steeper near day 40. (**D**) Hyperoxia on day 35 resulted in significantly steeper mortality trajectory in *per01* males compared to CS^p^ (p<0.001). Mortality trajectory in flies with restored per function (*{per01 {per+}*) was indistinguishable from CS^p^.
