# Supplementary material for: The circadian clock gene period extends healthspan in aging Drosophila melanogaster
Source: Aging (Albany NY). 2009 Nov 19;1(11):937–48. doi: 10.18632/aging.100103 (PMC2815745; doi:10.18632/aging.100103)
Supplement: Supplementary Table 1 — Mortality at age x (μx) is given as μx = aebx + c, where a is the baseline mortality rate (intercept), b is the age-dependent increase in mortality (slope), and c is the age-independent mortality. [file aging-01-937-s001.doc]

| **Treatment** | **Gompertz-Makeham parameters** | | | **Actual lifespan** | **Fitted lifespan** | **% Error**  **in lifespan** |
| --- | --- | --- | --- | --- | --- | --- |
|  | **a (intercept)** | **b**  **(slope)** | **c (constant)** |  |  |  |
|  | **MLE value** | **MLE value** | **MLE value** |  |  |  |
| **Normoxia CS^p^**  ***per^01^*** | 1.0 (10^-4^) | 0.1096 | 1.0 (10^-9^) | 61.5295 | 61.3032 | 0.2 |
|  | 2.0 (10^-4^) | 0.1225 | 1.0 (10^-9^) | 59.0313 | 59.1527 | 0.4 |
| **Hyperoxia CS^p^**  **day 5 *per^01^*** | 5.2 (10^-8^) | 0.2061 | 2.1 (10^-9^) | 60.4421 | 60.8754 | 0.05 |
|  | 5.5 (10^-7^) | 0.2387 | 5.0 (10^-9^) | 56.9486 | 56.479 | 0.29 |
| **Hyperoxia CS^p^**  **day 20 *per^01^*** | 1.0 (10^-5^) | 0.1366 | 2.1 (10^-9^) | 58.3614 | 58.2499 | 0.19 |
|  | 1.0 (10^-4^) | 0.1480 | 2.1 (10^-9^) | 51.3507 | 58.2382 | 0.22 |
| **Hyperoxia CS^p^**  **day 35 *per^01^***  ***per^01^{per^+^}*** | 2.8 (10^-6^) | 0.1770 | 2.1 (10^-9^) | 59.5641 | 59.3094 | 0.43 |
|  | 6.4 (10^-6^) | 0.1897 | 2.4 (10^-9^) | 47.8511 | 47.6659 | 0.39 |
|  | 2.6 (10^-6^) | 0.1710 | 2.1 (10^-9^) | 57.7429 | 57.3871 | 0.61 |
