# Supplementary material for: The circadian clock gene period extends healthspan in aging Drosophila melanogaster
Source: Aging (Albany NY). 2009 Nov 19;1(11):937–48. doi: 10.18632/aging.100103 (PMC2815745; doi:10.18632/aging.100103)
Supplement: Supplementary Table 2 — Values are Mean ± SEM of 3 separate bioreplicates. Three-way ANOVA with Bonferroni's post-hoc tests was performed for each tissue. Values with different superscripts shown in columns are significantly different at p<0.01. For comparison between genotypes (rows) for each treatment, * = p<0.05 and ** = p<0.001, † = p<0.03 ‡ = p<0.01. Comparison between treatments for each genotype showed significance at p<0.001 in all ages for heads, and on day 35 and 50 for bodies. [file aging-01-937-s002.doc]

| **Age (Days)/ Tissue** | **Normoxia** | | | **Hyperoxia** | | |
| --- | --- | --- | --- | --- | --- | --- |
| **Heads** | **CS^p^** | ***per^01^*** | ***per^01^*{*per^+^*}** | **CS^p^** | ***per^01^*** | ***per^01^*{*per^+^*}** |
| **5** | 5.8 ± 0.5^a^ | 7.6 ± 0.9^a^ |  | 14.5 ± 3.7^a^ | 19.2 ± 2.9^a^ |  |
| **20** | 15.6 ± 1.0^b^ | 17.4 ± 3.3^b^ |  | 36.6 ± 1.4^b^ | 47.9 ± 2.1^b**^ |  |
| **35** | 41.0 ± 2.4^c^ | 52.6 ± 0.5^c**^ | 38.6 ± 3.3^a^ | 55.1 ± 3.5^c^ | 72.1 ± 3.1^c**^ | 52.7 ± 3.2^a^ |
| **50** | 45.2 ± 3.5^c^ | 57.3 ± 4.1^c*^ | 41.3 ± 2.0^a^ | 61.1 ± 5.3^c^ | 87.6 ± 3.3^d**^ | 59.3 ± 3.0^a^ |
| **Bodies** |  |  |  |  |  |  |
| **5** | 2.7 ± 0.3^a^ | 4.3 ± 1.0^a^ |  | 4.5 ± 1.2^a^ | 5.7 ± 0.4^a^ |  |
| **20** | 7.9 ± 0.3^b^ | 9.6 ± 1.0^b^ |  | 9.8 ± 1.0^b^ | 12.6 ± 0.2^b†^ |  |
| **35** | 8.7 ± 1.4^b^ | 12.3 ± 3.0^b^ | 7.1 ± 2.0^a^ | 19.0 ± 2.2^c^ | 31.7 ± 4.2^c†^ | 18.3 ± 3.5^a^ |
| **50** | 19.0 ± 3.0^c^ | 28.4 ± 2.3^c†^ | 19.2 ± 1.5^b^ | 29.1 ± 3.8^d^ | 48.1 ± 5.1^d‡^ | 32.1 ± 2.0^b^ |
