# Supplementary material for: The circadian clock gene period extends healthspan in aging Drosophila melanogaster
Source: Aging (Albany NY). 2009 Nov 19;1(11):937–48. doi: 10.18632/aging.100103 (PMC2815745; doi:10.18632/aging.100103)
Supplement: Supplementary Table 3 — Values are Mean ± SEM of 3 separate bioreplicates. Three-way ANOVA with Bonferroni's post-hoc tests was performed for each tissue. Values in columns with different superscripts are significantly different at p<0.001. For comparison between genotypes (rows) for each treatment, † = p<0.03, Ψ = p<0.05, ** = p<0.001, *** = p<0.0001. Comparison between treatments for heads showed significant difference (p<0.01) at all ages for per01, and on day 35 and 50 for CSp. In case of bodies, comparison between treatments showed significance at p<0.01 on day 35 and 50 for both genotypes. [file aging-01-937-s003.doc]

| **Age (Days)/ Tissue** | **Normoxia** | | | **Hyperoxia** | | |
| --- | --- | --- | --- | --- | --- | --- |
| **Heads** | **CS^p^** | ***per^01^*** | ***per^01^*{*per^+^*}** | **CS^p^** | ***per^01^*** | ***per^01^*{*per^+^*}** |
| **5** | 0.02 ± 0.0^a^ | 0.02 ± 0.0^a^ |  | 0.04 ± 0.01^a^ | 0.05 ± 0.01^a^ |  |
| **20** | 0.2 ± 0.03^b^ | 0.3 ± 0.02^b†^ |  | 0.3 ± 0.01^b^ | 0.4 ± 0.01^b**^ |  |
| **35** | 0.45 ± 0.03^c^ | 0.5 ± 0.02^c^ | 0.41 ±0.05^a^ | 0.51 ± 0.04^c^ | 0.8 ± 0.02^cφ^ | 0.47 ± 0.2^a^ |
| **50** | 0.6 ± 0.0^d^ | 0.7 ± 0.02^d**^ | 0.65 ± 0.1^b^ | 0.7 ± 0.02^d^ | 0.9 ± 0.1^cφ^ | 0.72 ±0.3^b^ |
| **Bodies** |  |  |  |  |  |  |
| **5** | 0.14 ± 0.0^a^ | 0.14 ± 0.0^a^ |  | 0.14 ± 0.0^a^ | 0.15 ± 0.0^a^ |  |
| **20** | 0.24 ± 0.02^b^ | 0.3 ± 0.02^b^ |  | 0.32 ± 0.04^b^ | 0.4 ± 0.02^b^ |  |
| **35** | 0.52 ± 0.04^c^ | 0.6 ± 0.03^c^ | 0.58 ± 0.0^a^ | 0.54 ± 0.03^c^ | 0.7 ± 0.02^c**^ | 0.5 ± 0.5^a^ |
| **50** | 0.84 ± 0.02^d^ | 0.9 ± 0.04^d^ | 0.79 ± 0.5^b^ | 1.02 ± 0.04^d^ | 1.2 ± 0.01^d**^ | 0.9 ± 2.0^b^ |
