# Supplementary figures and images for: The circadian clock gene period extends healthspan in aging Drosophila melanogaster
Source: Aging (Albany NY). 2009 Nov 19;1(11):937–48. doi: 10.18632/aging.100103 (PMC2815745; doi:10.18632/aging.100103)

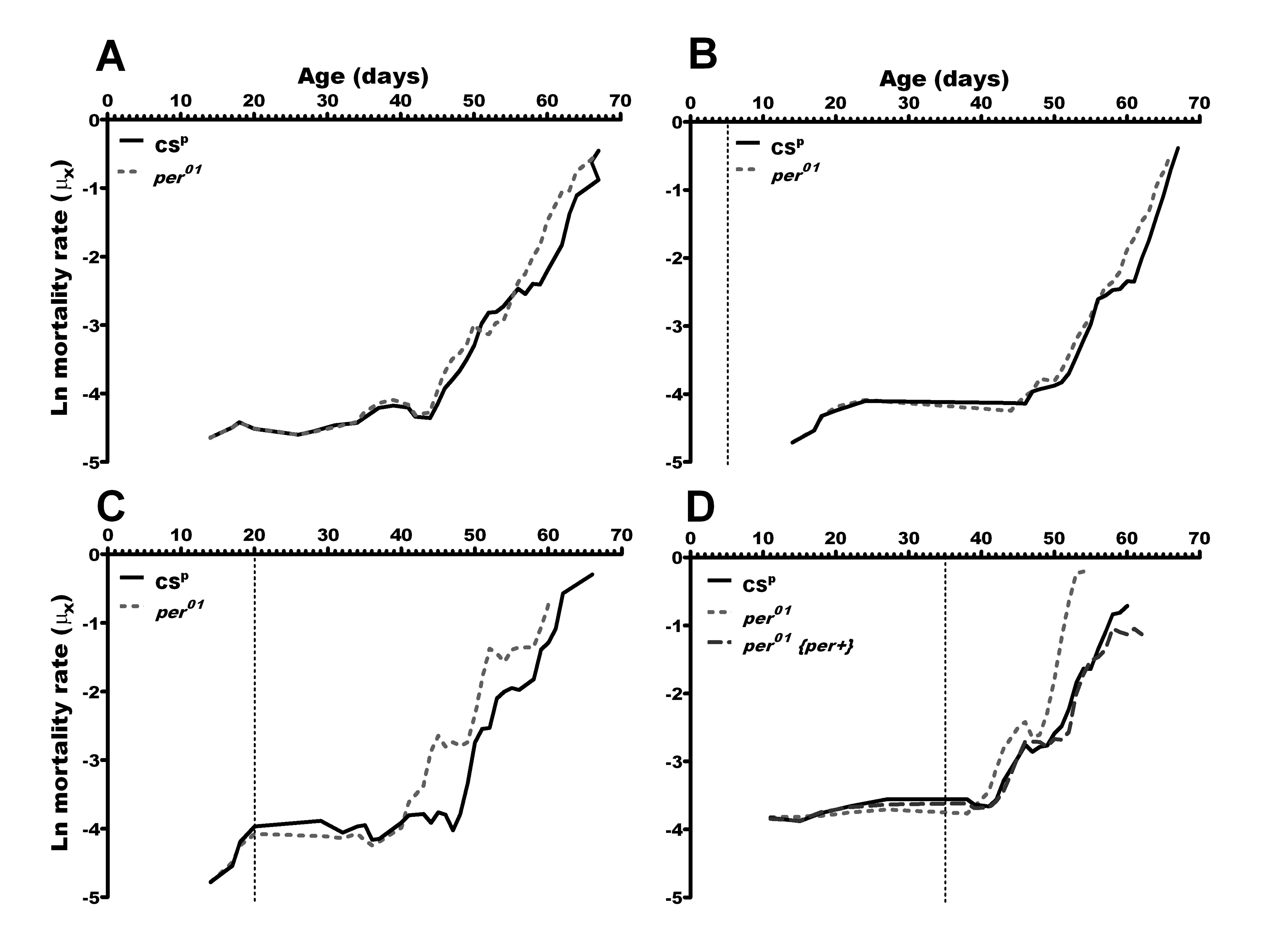

Supplement: Supplementary Figure 1 — Mortality trajectories were plotted using Gompertz-Makeham mortality parameters and smoothed using 2nd order smoothing of 5 neighbors. (A-B) Under normoxia and 24h hyperoxia on day 5 no significant difference in mortality trajectories was observed between CSp and per01 flies. (C) 24h hyperoxia on day 20 resulted in significantly different mortality trajectories (p<0.001), with mortality slope of per01 flies becoming steeper near day 40. (D) Hyperoxia on day 35 resulted in significantly steeper mortality trajectory in per01 males compared to CSp (p<0.001). Mortality trajectory in flies with restored per function ({per01 {per+}) was indistinguishable from CSp. [file aging-01-937-s001.tif]
